# Doxycycline-Induced Changes in Circulating MMP or TIMP2 Levels Are Not Associated with Skeletal-Related Event-Free or Overall Survival in Patients with Bone Metastases from Breast Cancer

**DOI:** 10.3390/cancers15030571

**Published:** 2023-01-17

**Authors:** Huijun Zhao, Gregory Pond, Demetrios Simos, Zhou Wang, Susan Robertson, Gurmit Singh, Lisa Vandermeer, Mark Clemons, Christina Lynn Addison

**Affiliations:** 1 Program for Cancer Therapeutics, Ottawa Hospital Research Institute, 501 Smyth Road, Ottawa, ON K1H 8L6, Canada; 2 Department of Oncology, McMaster University, 699 Concession Street, Hamilton, ON L8V 5C2, Canada; 3 Division of Medical Oncology, Department of Medicine, University of Ottawa, 451 Smyth Road, Ottawa, ON K1H 4M5, Canada; 4 Department of Pathology and Laboratory Medicine, University of Ottawa, 451 Smyth Road, Ottawa, ON K1H 4M5, Canada; 5 Department of Pathology and Molecular Medicine, McMaster University, 1280 Main Street West, Hamilton, ON L8S 4L8, Canada; 6 Department of Medicine and Biochemistry, University of Ottawa, 451 Smyth Road, Ottawa, ON K1H 4M5, Canada; 7 Department of Microbiology and Immunology, University of Ottawa, 451 Smyth Road, Ottawa, ON K1H 4M5, Canada

**Keywords:** doxycycline, breast cancer, bone metastasis, matrix metalloproteinase, MMP2, MMP9, TIMP2, SRE

## Abstract

**Simple Summary:**

The current study was a retrospective exploratory study evaluating whether circulating levels of known targets of doxycycline were altered following administration of doxycycline to bone metastatic breast cancer patients as an indicator of its expected on-target efficacy. Although we saw predicted changes in these proteins, namely MMP2, MMP9 and TIMP2 pre- and post-doxycycline, they were not prognostic for skeletal-related event-free or overall survival in this cohort. This is likely due in part to confounding effects of doxycycline administration on other cell types in the bone and effects of concurrent treatment regimens on these same target proteins. Although use of doxycycline in cancer patients remains an attractive modality, our findings suggest that studies assessing biomarkers of doxycycline efficacy should carefully consider putative confounding factors and account for this prospectively in the study design.

**Abstract:**

Doxycycline is often used as a promoter of inducible gene expression in preclinical models; however, it can also have direct effects on tumor growth and survival. This is due in part to its ability to inhibit cell invasion and regulate matrix metalloproteinase (MMP) expression. Given that doxycycline is also osteotropic, a clinical study to assess its effects on modulation of tumor progression or prevention of skeletal-related events (SRE) in patients with bone metastases from breast cancer (the Achilles trial) was undertaken. Patients received 100 mg of oral doxycycline twice daily for 12 weeks, with serum obtained at baseline and 4, 8 and 12 weeks post-initiation of doxycycline treatment. Exploratory analysis of the effects of doxycycline on circulating levels of MMP or tissue inhibitor of matrix metalloproteinase 2 (TIMP2) was performed in enrolled patients. Statistically significant associations were observed between MMP2, MMP9 and TIMP2 at baseline with significant associations maintained between absolute levels and changes in levels of MMP2 and TIMP2 at weeks 4–12 post initiation of doxycycline. Treatment with doxycycline generally resulted in decreases in MMP2 and MMP9 levels with concurrent upregulation of TIMP2 at 12 weeks post-initiation of doxycycline treatment. Despite this, we observed no association with the levels of any of these factors with either SRE-free or overall survival in this patient cohort. In summary, despite observing hypothesized effects of doxycycline administration on surrogate markers of its anti-tumor activity, measures of circulating levels of these biomarkers were not prognostic in this patient population.

## 1. Introduction

Despite originally being developed as an antibiotic to inhibit bacterial growth by blocking protein translation dependent on 30S ribosomes [1,2], the tetracycline analogue doxycycline also inhibits translation in mammalian cells through a similar process [3]. As such, doxycycline has been reported to have anti-tumor effects in a number of preclinical model systems [4,5,6,7] in part due to its ability to inhibit expression of known tumor-promoting factors such as matrix metalloproteinases (MMP) [7,8,9,10,11], angiogenic factors [11,12], and factors promoting cancer stem-like cell phenotypes [13,14,15,16]. This suggests that repurposing of doxycycline for cancer treatment could be beneficial in controlling tumor growth and progression.

Bone is the most common site of metastasis for estrogen receptor (ER)-positive breast cancers [17], and once breast tumor cells have spread to the bone, they disturb the balance of bone turnover to cause net bone loss through osteoclast-mediated bone degradation [18,19]. This bone loss can be associated with significant complications for patients, collectively called skeletal-related events (SRE), which include: the need for radiotherapy or surgery to alleviate skeletal complications, development of a pathological fracture, spinal cord compression and hypercalcemia of malignancy [20]. A number of therapeutic strategies designed to inhibit this net bone loss are currently used in the treatment of bone metastatic breast cancer patients. These include calcium-binding agents with high affinity for the bone such as the bisphosphonates (e.g., zoledronic acid) or agents that block the activity of one of the driving factors regulating bone degradation by osteoclast activity (e.g., denosumab). However, although these agents have been shown to benefit patients by preventing or prolonging the time to first SRE, they have had no effect on improving overall survival in patients [21,22,23]. Thus, more effective strategies to control bone metastatic tumor growth and prolong overall survival are urgently needed.

Similar to bisphosphonates, doxycycline also has an affinity for binding calcium and as a result accumulates in bony tissues [24]. Given its reported anti-tumor activities and potential to accumulate in the bone, doxycycline could have significant anti-tumor activity and control the growth of breast cancer bone metastases. Preclinical testing in breast cancer bone metastases models supported this notion and showed that administration of doxycycline in combination with the bisphosphonate zoledronic acid significantly inhibited tumor burden in the bone compared to untreated mice, which was concomitant with decreases in bone resorption and increases in bone formation [25]. Moreover, results from a small Phase I study suggested that patients with newly diagnosed bone metastases could benefit from administration of doxycycline as measured by decreased levels of circulating markers of bone turnover [26]. Based on these promising preclinical and early clinical findings, our team initiated a Phase II clinical study in bone metastatic breast cancer patients testing the effects of twice-daily oral administration of doxycycline, with the primary endpoint being assessment of palliative benefit of its inclusion to standard-of-care bone metastatic breast cancer therapy (The Achilles Study; ClinicalTrials.gov Identifier: NCT01847976). We hypothesized that given the role of doxycycline in modulating proteases that could contribute to osteolysis, which in turn leads to risk of SREs, its use in combination with bone-targeting agents (e.g., bisphosphonates or denosumab) could further inhibit the osteolytic process, resulting in decreased frequency of SRE or increased time to first SRE in treated patients. The primary results of this study have been previously reported in detail; however, no significant benefit to patients was observed [27]. As it remained possible that the doxycycline treatment failed to have relevant biological effects at the dosing regimen given, we sought to determine whether its affects on known targets could be confirmed in this patient cohort. We report herein on the effects of a 12-week cycle of doxycycline administration on the circulating levels of the known targets MMP2, MMP9 and their inhibitor TIMP2 in breast cancer patients with bone metastases and their further associations with relevant clinical outcomes.

## 2. Methods

### 2.1. Trial Design and Study Population

The Achilles study (ClinicalTrials.gov Identifier: NCT01847976) and its primary endpoint analysis are detailed elsewhere [27]. Briefly, Achilles enrolled 37 metastatic breast cancer patients with radiologically or biopsy-confirmed bone metastases, established on standard breast cancer therapies, who had previously received a minimum of 3 months of bone-targeted agent therapy (e.g., bisphosphonates or denosumab) and who met additional eligibility criteria in the trial protocol approved by the Ottawa Health Science Network Research Ethics Board (approved protocol 20120543-01H). Enrolled patients received 100 mg doxycycline orally twice daily for a period of 12 weeks, and self-reported drug compliance and bone pain were collected using validated pain questionnaires (Brief Pain Inventory (BPI) [28,29] and Functional Assessment of Cancer Therapy-Bone Pain (FACT-BP)) [30,31]. Patients were also assessed for frequency of skeletal-related events (SRE) prior to enrollment and subsequent to study completion with SRE defined as the occurrence of radiotherapy or surgery to the bone, pathological fractures, spinal cord compression, or hypercalcemia of malignancy [20]. Breast cancer-specific overall survival (OS) was defined as time from study completion until death due to breast cancer. While the original approved protocol allowed for 2 years of follow up for patients, the approval was extended to 5 years of patient follow up for the current study to capture survival and additional SRE data at 1, 2 and 5 years post-study entry.

### 2.2. Serum Sampling

Serum was collected early in the morning following an overnight fast at baseline study entry and at 4, 8 and 12 weeks post-initiation of doxycycline treatment. Samples were allowed to clot and were centrifuged at 4 °C for 10 min at 3400 RPM, with cleared serum transferred to multiple cryovials and stored at −80 °C until subsequently analysis. For analysis of circulating factors, frozen serum was thawed once and analyzed by ELISA directly as described below.

### 2.3. ELISA Analysis

Human Quantikine 96-well plate-based ELISA kits for MMP2 (cat# MMP200), MMP9 (cat# DMP900), and TIMP2 (cat#DTM200) were purchased from R&D Systems (Minneapolis, MN, USA). Serum samples were thawed on ice and measured in duplicate technical replicates for each sample as described in the manufacturer’s protocol. Absorbance of each well was measured at 450 nm in the Multiskan Ascent plate reader (Thermofisher, Ottawa, ON, Canada). Sample concentration for each protein of interest was calculated following interpolation of a four-parameter logistic curve generated by the Multiskan Ascent software v1.3.2 using results obtained from increasing known concentrations of each protein. For the purpose of statistical analysis, duplicate measures were included as independent measures. Assay sensitivities were as follows: MMP2 ~ 0.1 ng/mL, MMP9 ~ 0.2 ng/mL, and TIMP2 ~ 0.1 ng/mL.

### 2.4. Statistical Analyses

Descriptive statistics were used to summarize patient characteristics, biomarker targets and clinical outcomes. Biomarker targets were summarized at each time point evaluated (i.e., baseline and weeks 4, 8, 12). Percent change in biomarker levels was calculated as (week X value—baseline value)/baseline value × 100%. Spearman correlation coefficient testing was used to determine the association between different biomarker values and between the change in different biomarker values from baseline to weeks on study. Potential prognostic ability of biomarker levels for SRE-free or overall survival, measured from the day of first treatment to event or last date the patient was confirmed to be event-free, was assessed using Cox proportional regression analyses. Multivariable models were constructed adjusting for clinical variables found to be associated with outcome. All statistical analyses were performed using SAS version 9.2 (SAS Institute, Cary, NC, USA), with two-sided testing and a *p* value of 0.05 or less considered statistically significant.

## 3. Results

### 3.1. Patient Cohort in the Achilles Study

Between April 2013 and May 2015, patients consented and were enrolled in the Achilles study. Detailed description of patient enrollment and patient baseline characteristics have been previously reported [27]. Patient characteristics relevant to the current study are presented in Table 1. At study enrollment, the median patient age was 59 (range 41–88 years old), and patients generally had a good performance status with ~60% of patients with an ECOG status of 1. The median duration of bone metastases was 13 months (range 4–67 months), and the median duration of treatment for bone metastases prior to study entry was 10 months (range 3–67 months). More than half the patients (54%) had experienced SRE prior to study enrollment. All patients were on a bone-targeting agent at the time of enrollment, including pamidronate (~42%), zoledronic acid (~53%) or denosumab (~6%). All patients were also on concurrent systemic treatments, including chemotherapy (~42%), chemotherapy plus trastuzumab (~6%) and endocrine therapy (~53%). Of the enrolled patients, 28 (~76%) completed the 12-week doxycycline regimen with the remaining patients coming off study due to disease progression (*n* = 3), adverse events (*n* = 5) or unknown reasons (*n* = 1). Almost 90% of enrolled patients experienced at least one SRE (which in this cohort were all due to need for radiotherapy or pathological bone fractures) over a 5-year follow-up period after doxycycline treatment, and the 5-year overall survival rate was 27% in this cohort.

### 3.2. Levels of Circulating Putative Biomarkers over Time

Putative biomarker levels measured in serum are presented in Table 2. Median levels of all markers appeared to be relatively stable across all weeks measured when assessed in the entire cohort; however, the range for MMP9 levels tended to be larger compared to MMP2 or TIMP2, and its range varied more substantially across time points. Similarly, the range of changes in MMP9 was also larger compared to that observed for changes in MMP2 and TIMP2. Spearman correlation coefficients were used to assess relationships between putative biomarkers and indicated strong positive associations (ρ > 0.60) between MMP2 and TIMP2 at all time points examined (Table 3). Although a strong negative association (ρ = −0.60) was also observed between MMP9 and TIMP2 at baseline, this association was not apparent at any of the assessed time points post-initiation of doxycycline (Table 3).

Percentage changes of putative biomarkers in individual patients from baseline to each week on doxycycline trended for decreased MMP2 levels compared to baseline in patients on doxycycline at weeks 4 and 8, and decreased MMP9 levels compared to baseline were observed in patients on doxycycline at weeks 8 and 12 (Table 2). In contrast, TIMP2 levels were found to be increased in patients on doxycycline at week 12. Spearman correlation coefficient analysis also suggested strong (for weeks 4 and 8) or moderate (week 12) associations with the percentage changes in MMP2 and TIMP2 biomarker levels from baseline to on doxycycline (Table 4).

### 3.3. Association of Putative Biomarkers with SRE-Free or Overall Survival

Cox regression analysis was used to identify any potentially prognostic associations between clinical parameters or biomarker levels and SRE-free or overall survival in doxycycline-treated patients. Only higher ECOG status at baseline (HR 2.52, 95% CI (1.23, 5.17), *p* = 0.012) and previous SRE prior to study enrollment (HR 2.21, 95% CI (1.1, 4.45), *p* = 0.026) were found to significantly prognosticate SRE-free survival in univariate analysis. Although trends for baseline MMP2 and TIMP2 levels and change in MMP2 levels from baseline to week 8 with SRE-free survival were observed, these did not reach statistical significance (Table 5). Of note, after adjusting for baseline ECOG status, no significant associations with SRE-free survival were observed for any other variable.

Only ECOG status was found to be significantly associated with overall survival in this patient cohort (HR 2.91, 95% CI (1.34, 6.33), *p* = 0.007; Table 6). No other factor was statistically prognostic for overall survival when ECOG status was adjusted for as a confounding factor. There was a potential for a survivor bias when looking at the change in biomarker values from baseline to weeks 4, 8 or 12. However, it was determined as negligible given that only one patient had an event before the end of week 12; thus, the change in biomarker value was considered as a ‘baseline’ measurement for the purposes of this analysis.

## 4. Discussion

Doxycycline has been previously reported to have anti-tumor activity in multiple tumor models [5,7,32] including breast cancer [4,6,8,25,33]. It has also been shown to suppress expression of MMP2 and MMP9 [8,10,11,32], thus suggesting that measurement of these two MMP proteins could be a biological readout of doxycycline activity. TIMP2 is a known inhibitor of MMP activity and tumor growth [34,35,36,37,38] that has been shown to be reciprocally downregulated with MMP2 upregulation [39]. As such, we evaluated circulating levels of MMP2, MMP9 and TIMP2 as indicators of doxycycline activity in patients enrolled in the Achilles trial. As hypothesized, we generally observed decreases in MMP2 and MMP9 levels and increases in TIMP2 levels in patients following doxycycline administration, suggesting that the dosing regimen reached effective levels in patients. We also observed a significant association between MMP2 and TIMP2 levels as would be predicted based on previous literature suggesting their reciprocal co-regulation [39]. Despite this, we did not observe any significant relationship with the clinical parameters of SRE-free or overall survival.

Previous preclinical work demonstrated that doxycycline was able to significantly inhibit bone metastatic tumor burden in the MDA-MB-231 xenograft model in part due to its osteotropism and ability to inhibit MMP secretion and activity [4]. It was also shown to significantly inhibit bone metastases when used in combination with the bone targeting agent zoledronic acid in xenograft models [25]. Thus, the Achilles study enrolled patients with metastatic breast cancer with radiologically confirmed bone metastases who were receiving anticancer treatment and who were on bone-targeted therapy (bisphosphonate or denosumab) for at least 3 months prior to study enrollment. Enrolled patients received 100 mg doxycycline orally twice per day for 12 weeks, with the hypothesis that doxycycline administration in combination with ongoing bone-targeted therapy could provide added palliative benefit to patients via its ability to inhibit tumor progression and bone osteolysis. The effects of doxycycline administration on pain outcomes and bone turnover markers in the Achilles study have been previously reported [27]; however, no significant benefit was observed with respect to these clinical outcomes. The Achilles study used a similar doxycycline dosing regimen to that previously reported in a Phase I study in 17 patients with newly diagnosed metastatic breast cancer to the bone, which observed trends for decreased bone turnover as measured by the markers N-telopeptide and bone-specific alkaline phosphatase (BSAP) following doxycycline administration [26]. Additional prognostic markers or markers of doxycycline activity were not reported in this study [26]. Patients in this study were not receiving additional concurrent treatments at the time of doxycycline administration [26]. Unlike this previous study, it is possible that benefits in bone turnover indicators were not observed in the Achilles cohort due to differences in enrollment eligibility, with metastatic patients enrolled in the Achilles study being more advanced and already established and maintained on anticancer treatment as well as receiving bone-targeting agents for a minimum of 3 months prior to doxycycline treatment (bone-targeting agent treatment duration ranged from 3 to 67 months prior to study entry; Table 1). It is also possible that these more advanced heavily pretreated metastatic patients may be less responsive to the beneficial effects of doxycycline. More recently, it has been shown that pre-operative “window” administration of twice daily 100 mg doxycycline for 14 days to newly diagnosed breast cancer patients was effective in reducing the breast cancer stem cell population (measured by both CD44 and ALDH1) [40], suggesting it may provide more clinical benefit in prevention of metastases as opposed to treatment of metastases. This however cannot be confirmed, as the study was designed as a ‘window of opportunity’ study to assess doxycycline effects on tumor cells, with patients receiving curative therapy thereafter. Lastly, it is also possible that effects were not observed due to the relatively small sample size of this study.

The Achilles study was designed as a single-arm study and was powered for the primary objective of assessing pain control using validated pain questionnaires and tumor-induced bone turnover using biomarkers such as C-telopeptide (CTx) and bone-secreted alkaline phosphatase (BSAP) [27]. As these were the primary objectives, enrolled patients were allowed to remain on the current therapy being received at time of enrollment, as it is established that markers of bone turnover can be affected by changes in systemic therapy [41,42]. It thus remains possible that the circulating levels of the MMP and TIMP biomarkers were affected by previous and concurrent systemic treatments and their effects on either tumor or non-tumor tissues in the bone. All enrolled patients were on bone-targeting agents at study entry, and it has been shown in vitro that bone-targeting agents (including clodronate, pamidronate and zoledronic acid) can inhibit tumor cell expression of MMP2 and MMP9 [43]. However, their effects on other tissue types can be variable. For example, it has been previously shown that pamidronate can induce while clodronate can inhibit MMP9 gene expression in treated human monocyte/macrophage cells in vitro [44]. Similarly, patients could also be taking anti-estrogens such as tamoxifen or fulvestrant, both of which have been shown to increase MMP2 and MMP9 secretion from breast tumor cells [45,46]. As patients were required to be on bone-targeting agents to be eligible to enroll in the current study, and were allowed to remain on other systemic therapies (which included chemotherapy or endocrine therapy regimens in this cohort) during doxycycline administration, it is likely that despite observing the expected doxycycline-induced changes in MMPs and TIMP2, their association with patient outcome measures was likely confounded by alterations in their levels at baseline and throughout the study due to these previous and concurrent treatment regimens. Assessment of circulating levels of MMPs or TIMP2 in patients enrolled in Achilles was also exploratory in nature, and thus it remains possible that this study is insufficiently powered to detect their prognostic associations, which is a significant limitation of the current study. The single-arm design of this study is also a limitation, as it would have been of interest to assess whether changes in MMPs or TIMP2 in doxycycline treated patients was altered compared to those on similar concurrent standard of care treatments alone. However, given that doxycycline administration appeared to have no beneficial effect on bone metastasis progression in this patient cohort as indicated by the bone-turnover marker measures such as CTx [27], it is perhaps not surprising that we failed to demonstrate any prognostic associations with the other circulating markers described here.

Although circulating levels of MMPs and TIMP2 suggested effective doxycycline administration, it is possible that levels of these markers specifically in the bone microenvironment would be a more accurate measure of doxycycline activity and effects on bone tumor growth given that a variety of different cell types throughout the body can also produce and contribute to levels of these factors in circulation. Our original objective was to also assess MMP and TIMP levels in tumor tissue obtained from the bone metastatic microenvironment at baseline and at 12 weeks following doxycycline administration. While iliac crest bone biopsies were obtained from 36 of the 37 patients enrolled in Achilles at baseline, serial biopsy at week 12 was only obtained in 25 of 37 patients [27]. Unfortunately, only 6 of the 36 baseline and only 3 of the 25 biopsies taken at week 12 were positive for tumor cells. Only one of these patients had paired baseline and week-12 biopsies positive for tumor cells, making any correlative assessment of pre and post-doxycycline target measures in tissue samples impossible.

## 5. Conclusions

While use of doxycycline administration in this patient cohort was not beneficial, its use clinically remains attractive due to its already established modes of action, toxicity profile and government approvals for use in patients. Our study, however, suggests that in order to accurately evaluate potential prognostic markers or markers of drug efficacy in response to doxycycline administration, one must carefully design the study to consider the effects of doxycycline on other cell types in the body in addition to the effects of other therapeutic agents on the putative doxycycline biomarkers to avoid confounding results.

## Figures and Tables

**Table 1:** Characteristics and outcomes.

Characteristics or Outcome	*N*		All Patients
*N*			**37**
Demographics			
Age	37	Median (IQR), Range	59 (54–65), 41–88
		Mean (std dev)	60.1 (10.1)
ECOG Status at Baseline	37		N (%)
		1	22 (59.5)
		2	14 (37.8)
		3	1 (2.7)
Duration of Bone Metastasis (months)	35	Median (IQR), Range	13 (8–22), 4–67
Time from Primary to Metastases (months)	37	Median (IQR), Range	3 (0–108), 0–312
Bone Therapy Duration (months)	37	Median (IQR), Range	10 (7–20), 3–67
Vitamin D at baseline	37	Median (IQR), Range	91 (72–120), 48-185
PTH at baseline	37	Median (IQR), Range	5.0 (3.4–7.1), 1.7–18.3
Therapy Type	36		N (%)
		Chemotherapy	15 (41.7)
		Chemotherapy + Trastuzumab	2 (5.6)
		Endocrine Therapy	19 (52.8)
Previous SRE Radiation for Bone Pain	37	(number of occurrences)	N (%)
		0	18 (48.7)
		1	15 (40.5)
		2	1 (2.7)
		3	2 (5.4)
		4	0
		5	1 (2.7)
Previous SRE Radiotherapy Preventative	37	N (%)	15 (40.5)
Past SRE Bone Surgery	37	N (%)	6 (16.2)
Past SRE Hypercalcaemia	37	N (%)	3 (8.1)
Past SRE Spinal Cord Compression	37	N (%)	6 (16.2)
Past SRE Path Fracture	37	(number of occurrences)	N (%)
		0	26 (70.3)
		1	8 (21.6)
		2	3 (8.1)
Previous SRE Total	37	(number of occurrences)	N (%)
		0	17 (46.0)
		1	3 (8.1)
		2	3 (8.1)
		3	3 (8.1)
		4	5 (13.5)
		5	4 (10.8)
		6	0
		7	2 (5.4)
Outcomes			
Study Completion Status	37	Completed Study	28 (75.7)
		Withdrew due to PD	3 (8.1)
		Withdrew due to AE	5 (13.5)
		Unknown	1 (2.7)
SRE Type	19	Fracture	3
		Radiation	16
Time to SRE or Death	37	N (%) Events	33 (89.2)
		Median (95% CI)	16.8 (10.5, 27.2)
		1-Year SRE-Free Rate (95% CI)	62.2 (44.6, 75.6)
		2-Year SRE-Free Rate (95% CI)	43.2 (27.2, 58.3)
		5-Year SRE-Free Rate (95% CI)	16.2 (6.6, 29.6)
Time to SRE or Death	37	N (%) Events	32 (86.5)
		Median (95% CI) OS	33.8 (21.5, 43.9)
		1-Year OS (95% CI)	81.1 (64.4, 90.5)
		2-Year OS (95% CI)	59.5 (42.0, 73.2)
		5-Year OS (95% CI)	27.0 (14.1, 41.8)

**Table 2:** Circulating levels of putative biomarkers.

**Absolute Circulating Levels**			
**Biomarker**	**N**	**Timepoint**	**Median ng/mL (Range)**
MMP2	34	Baseline	262 (125–401)
	31	Week 4	256 (139–359)
	29	Week 8	263 (128–477)
	26	Week 12	274 (157–384)
MMP9	33	Baseline	237 (33–801)
	30	Week 4	276 (39–931)
	29	Week 8	261 (108–665)
	25	Week 12	262 (72–593)
TIMP2	34	Baseline	120 (86–178)
	31	Week 4	122 (85–165)
	29	Week 8	117 (80–174)
	26	Week 12	128 (87–226)
**Change in Circulating Levels from Baseline**			
**Biomarker**	**N**	**Baseline to Time point**	**Percentage Change (Range)**
MMP2	31	Week 4	−3.8 (−38.7, 77.6)
	29	Week 8	−3.5 (−29.4, 76.5)
	26	Week 12	0.9 (−23.6, 90.9)
MMP9	29	Week 4	4.9 (−75.0, 851.1)
	28	Week 8	−4.0 (−66.4, 682.0)
	25	Week 12	−6.4 (−64.5, 950.4)
TIMP2	31	Week 4	−0.3 (−19.5, 32.1)
	29	Week 8	−0.5 (−26.2, 24.8)
	26	Week 12	4.1 (−32.4, 87.9)

**Table 3:** Spearman correlation coefficients between different markers.

Markers	MMP9	TIMP2
Baseline		
MMP2	−0.48 **	0.77 **
MMP9		−0.60 **
Week 4		
MMP2	−0.12	0.81 **
MMP9		−0.16
Week 8		
MMP2	0.27	0.80 **
MMP9		0.32
Week 12		
MMP2	0.38	0.61 **
MMP9		−0.18

** Statistically significant at the α = 0.01 level.

**Table 4:** Spearman correlation coefficients between % change in different markers.

Markers	MMP9	NTx	TIMP2	Week 4	Week 8	Week 12
At Week 4				Baseline with Later Values		
MMP2	0.21	−0.07	0.46 **	0.75 **	0.73 **	0.50 **
MMP9		−0.04	0.19	0.44 *	0.47 *	0.45 *
TIMP2				0.71 **	0.74 **	0.59 **
At Week 8						
MMP2	0.39 *	−0.16	0.67 **			
MMP9		−0.35	0.42 *			
At Week 12						
MMP2	0.43 *	0.02	0.42 *			
MMP9		−0.05	0.05			

* Statistically significant at the α = 0.05 level. ** Statistically significant at the α = 0.01 level.

**Table 5:** Cox regression for prognostic factors of SRE-free survival.

Factor	*N*	Comparator	HR (95% CI)	*p*-Value
Age	37	/year	1.01 (0.98, 1.05)	0.48
ECOG Status at Baseline	37	2/3 vs. 1	2.52 (1.23, 5.17)	0.012 *
Duration of Bone Mets	35	/month	1.00 (0.98, 1.02)	0.94
Time from Primary to Metastases	37	/month	1.00 (1.00, 1.01)	0.70
Bone Therapy Duration (months)	37	/month	1.00 (0.97, 1.02)	0.68
Vitamin D at baseline	37	/unit	0.99 (0.98, 1.00)	0.12
PTH at baseline	37	/unit	1.06 (0.95, 1.19)	0.28
Past SRE Total	37	≥1 vs. 0	2.21 (1.10, 4.45)	0.026 *
Baseline MMP2	34	/100 unit	1.50 (0.86, 2.64)	0.16
Baseline MMP9	33	/100 unit	0.91 (0.73, 1.14)	0.42
Baseline TIMP2	34	/100 unit	4.48 (0.80, 25.25)	0.089
% Change in MMP2	31	To week 4	1.01 (0.99, 1.02)	0.33
	29	To week 8	6.19 (0.74, 51.92)	0.093
	26	To week 12	1.00 (0.99, 1.02)	0.74
% Change in MMP9	29	To week 4	1.00 (1.00, 1.00)	0.47
	28	To week 8	1.00 (1.00, 1.00)	0.44
	25	To week 12	1.00 (1.00, 1.00)	0.61
% Change in TIMP2	31	To week 4	0.99 (0.96, 1.03)	0.59
	29	To week 8	1.01 (0.97, 1.05)	0.68
	26	To week 12	0.98 (0.96, 1.01)	0.17

* Statistically significant at the α = 0.05 level in univariate analysis. After adjusting for ECOG status, no other factor was statistically significant.

**Table 6:** Cox regression for prognostic factors of overall survival.

Factor	*N*	Comparator	HR (95% CI)	*p* Value
Age	37	/year	1.01 (0.98, 1.05)	0.54
ECOG Status at Baseline	37	2/3 vs. 1	2.91 (1.34, 6.33)	0.007 **
Duration of Bone Mets	35	/month	1.00 (0.97, 1.02)	0.74
Time from Primary to Metastases	37	/month	1.00 (1.00, 1.01)	0.76
Bone Therapy Duration (months)	37	/month	0.99 (0.97, 1.02)	0.53
Vitamin D at baseline	37	/unit	0.99 (0.98, 1.00)	0.10
PTH at baseline	37	/unit	1.09 (0.96, 1.22)	0.17
Past SRE Total	37	≥1 vs. 0	1.40 (0.69, 2.83)	0.35
Baseline MMP2	34	/100 unit	1.34 (0.74, 2.41)	0.33
Baseline MMP9	33	/100 unit	0.95 (0.75, 1.21)	0.67
Baseline TIMP2	34	/100 unit	2.95 (0.54, 16.12)	0.21
% Change in MMP2	31	To week 4	1.01 (0.99, 1.02)	0.39
	29	To week 8	5.89 (0.55, 63.31)	0.14
	26	To week 12	1.00 (0.98, 1.02)	0.85
% Change in MMP9	29	To week 4	1.00 (1.00, 1.00)	0.57
	28	To week 8	1.00 (1.00, 1.00)	0.60
	25	To week 12	1.00 (1.00, 1.00)	0.79
% Change in TIMP2	31	To week 4	0.98 (0.95, 1.02)	0.31
	29	To week 8	1.01 (0.96, 1.06)	0.73
	26	To week 12	0.98 (0.96, 1.01)	0.15

** Statistically significant at the α = 0.01 level in univariate analysis. After adjusting for ECOG status, no other factor was statistically significant.

## Data Availability

The data are not publicly available due to privacy concerns related to the small sample size. The de-identified dataset is available upon request and approval by the Ottawa Hospital Science Network Research Ethics Board.
